# Influence of socioeconomic disparities, temperature and humidity in kidney stone composition

**DOI:** 10.1590/2175-8239-JBN-2019-0206

**Published:** 2020-07-20

**Authors:** Tamara da Silva Cunha, Adrian Rodriguez, Ita Pfeferman Heilberg

**Affiliations:** 1 Universidade Federal de São Paulo, Escola Paulista de Medicina, Divisão de Nefrologia, São Paulo, SP, Brazil.; 2 Universidade Federal do Rio de Janeiro, Divisão de Nefrologia, Rio de Janeiro, RJ, Brazil.; 3 Universita Cattolica del Sacro Cuore, Department of Medical Sciences, Rome, Italy.

**Keywords:** Kidney Calculi, Urolithiasis, Stone composition, Stone analysis, Cálculo renal, Urolitíase, Composição de cálculos renais, Análise cristalográfica

## Abstract

**Introduction:**

Large variations in demographic, economic and environmental factors might influence the worldwide distribution of urolithiasis, but scarce data are available concerning their associations with stone composition. We aimed to evaluate the frequency and composition of kidney stones and their associations with temperature, humidity, and human development index (HDI).

**Materials and Methods:**

A total of 1,158 stones from distinct patients (47±14 years old, male/female 2:1) were included. The mean annual temperature and relative humidity of each town were considered separately.

**Results:**

Calcium oxalate monohydrate (COM) was disclosed in 38.8% of patients, calcium oxalate dihydrate (COD) in 22.1%, mixed COD/apatite in 9.4%, pure apatite in 1.9%, brushite in 1.8%, struvite in 8.3%, pure uric acid in 11.1%, mixed uric acid/COM in 5.6%, and cystine/rare types in 0.8%. Mean HDI of all pooled cities was 0.780±0.03. However, people living in HDI<0.800 regions had twice the odds of having a struvite stone versus those living in HDI>0.800 (OR=2.14, 95% CI 1.11-4.11). Furthermore, a progressive increase in the struvite stones frequency from 4.5 to 22.8% was detected from HDI>0.800 through HDI<0.700. No significant difference for other stone types was disclosed. Separate logistic regression models assessed the association of each stone composition with gender, temperature, humidity and HDI as covariates.

**Conclusion:**

Patients living in low HDI areas are more prone to develop struvite stones, possibly due to lower access to healthcare. Temperature and humidity did not represent a specific risk factor for any stone type in the present sample.

## Resumo

**Introdução:**

Grandes variações em fatores demográficos, econômicos e ambientais podem influenciar a distribuição mundial da urolitíase, mas há muito pouco dado disponível sobre suas associações com a composição do cálculo renal. Nosso objetivo foi avaliar a frequência e composição dos cálculos renais e suas associações com temperatura, umidade e índice de desenvolvimento humano (IDH).

**Materiais e Métodos:**

Foram incluídos 1.158 cálculos de pacientes distintos (47 ± 14 anos, masculino / feminino 2:1). A temperatura média anual e a umidade relativa de cada cidade foram consideradas separadamente.

**Resultados:**

O oxalato de cálcio monohidratado (COM) foi detectado em 38,8% dos pacientes; oxalato de cálcio dihidratado (COD) em 22,1%; mistos de COD/apatita em 9,4%; apatita pura em 1,9%; brushita em 1,8%; estruvita em 8,3%, ácido úrico puro em 11,1%; mistos de ácido úrico /COM em 5,6% e cistina/tipos raros em 0,8%. O IDH médio de todas as cidades em conjunto foi de 0,780 ± 0,03. No entanto, indivíduos que vivem em regiões com IDH <0,800 apresentaram duas vezes a razão de chances de ter cálculo de estruvita do que aqueles que vivem em cidades com IDH > 0,800 (OR = 2,14; IC 95% 1,11-4,11). Além disso, um aumento progressivo na frequência de cálculos de estruvita de 4,5 para 22,8% foi detectado em IDH> 0,800 até IDH <0,700. Não foi observada nenhuma diferença significante para outros tipos de cálculos. Modelos separados de regressão logística foram utilizados para avaliar a associação de cada tipo de cálculo com gênero, temperatura, umidade e IDH como covariáveis.

**Conclusão:**

Pacientes que vivem em áreas com baixo IDH são mais propensos a desenvolverem cálculos de estruvita, possivelmente devido ao menor acesso à assistência médica. A temperatura e a umidade não representaram um fator de risco específico para qualquer tipo de cálculo na presente amostra.

## Introduction

Over the two past decades, the prevalence of urolithiasis has increased worldwide^1^^-^ 3. In the United States, it has markedly changed, almost doubling the prevalence during the last twenty years from 5 to 9%^4^^,^ 5. The reasons for this increase are still unclear, but changes in dietary habits affecting both urinary biochemical parameters and stone composition could represent causal factors^6^^,^ 7. To support this information, recent epidemiological data by Ferraro et al.^8^ examining cohorts including mostly calcium stone formers revealed that body mass index (BMI), sugar sweetened beverage intake, lower fluid intake, DASH (Dietary Approaches to Stop Hypertension) as well as calcium intake represented the most important modifiable factors for kidney stone prevention. The proportion of the less frequent uric acid stone within all stone formers also increased in the United States from 7 to 14% from 1980 to 2015, possibly ascribed to a higher BMI, older age, lower urinary pH, and increasing prevalence of diabetes and metabolic syndrome^9^. However, several other factors may be involved in the formation of kidney stones.

More than 100 chemical components have been identified within kidney stones, resulting in different etiologies regarding stone development and growth^10^. Besides dietary patterns, other contributing factors such as demographic, economic and environmental factors, e.g. climate (including temperature and humidity levels) may account for the increase in urolithiasis across the world. However, there are scarce data investigating their association with kidney stone composition^11^. Brazil, given its continental size and huge coastal extension, possesses five different climates with a large range of temperature and humidity levels as a result of the territorial extension through the tropics. Moreover, a wide variation of human development indexes (HDI) is observed across the country’s cities due to vast social contrasts and economic diversity, rendering unequal the access to the healthcare. Altogether, these assumptions have prompted us to examine the frequency and composition of kidney stones across different areas of the country and their relationships with demographic characteristics, climate, and HDI of each region.

## Materials and Methods

This retrospective study was carried out using collected urinary stones (passed spontaneously or after removal procedures) from different regions of Brazil, previously sent by mail for physical analysis between January 2017 and December 2018. Only the first submitted stone from each patient was examined. The current databases contained demographic information such as age, gender, report of previous episodes of urinary tract infections (UTI) in the last year, and the ZIP-code (providing geographic location). The study was approved by the Ethics Committee of the Universidade Federal de São Paulo (CEP-UNIFESP 0594/2018).

Stones physical analysis was performed, and morphologic examination and classification of the renal stone surface and section were combined with infrared spectroscopy (IR) to classify renal stones and identify the different crystalline phases according to European criteria^12^. All calculi were examined by the same investigator (TDSC) using stereoscope Opton TNG 01B and infrared spectrometer FT-IR Alpha (Bruker, Germany).

The mean annual temperatures and humidity levels from each city were obtained from the National Institute of Meteorology in Brazil (http://www.inmet.gov.br). The HDI of each city was obtained from the website according to the zip code from where each stone was acquired (http://atlasbrasil.org.br).

Brazilian climate is typically divided by the National Institute of Meteorology into: equatorial, tropical, humid tropical, semi-arid, and humid subtropical. For the current analysis the main climatic components (temperature and humidity) were considered separately, and each city classified according to the mean annual temperature (<20°C, 20-25°C, >25°C) and mean annual relative humidity (dry or humid). A cut-off level of 0.800 for the HDI was considered very high (http://hdr.undp.org/en/content/human-development-index-hdi), according to the Human Development Report Office - UN Development Program, p. 22-25, September 2018. The main outcome was kidney stone composition according to temperature, humidity, and HDI.

### Statistical analysis

Descriptive statistical analysis was performed, and continuous variables were reported as means with standard deviations, while categorical variables were reported as counts or percentages. For comparisons of categorical variables between groups, Pearson chi-squared test was used. A one-way analysis of variance was used to assess differences in continuous variables across multiple groups, with subsequent pairwise comparisons of means using Tukey-Kramer test. Logistic regression was performed to assess the association among stone composition and covariates (gender, temperature, humidity, and HDI). Separate logistic regression models were used with one model for each stone type; odds ratios (OR) with 95% confidence intervals (CIs) were calculated. SPSS Statistics for Windows, version 25 (IBM Corp) was used for all statistical analyses. The value of p<0.05 was considered statistically significant unless otherwise specified.

## Results

A total of 1,158 stones from distinct patients aged between 15 and 83 years with a male:female ratio of 2:1, and 47±14 years old (y/o) on average. The distribution of stone types and ages was as follwos: calcium oxalate monohydrate (COM) in 38.8% of patients (49±13 y/o), calcium oxalate dihydrate (COD) in 22.1% (43±14 y/o), mixed COD/apatite in 9.4% (44±14 y/o), pure apatite in 1.9% (46±15 y/o), struvite in 8.3% (44±14 y/o), brushite in 1.8% (41±12 y/o), pure uric acid in 11.1% (54±12 y/o), and mixed uric acid/COM stones in 5.6% (54±13 y/o). Cystine and other rare types represented only 0.8% of all stones. Seventy-eight percent of the patients reported recurrence and 69% of the total sample was obtained from surgical approach. Among different stone types, the mean age was significantly higher only in the two groups containing uric acid as the major component compared to all other groups (p<0.001). The mean HDI of all cities was 0.780±0.03 (range 0.610 - 0.850). The stone analysis according to gender distribution is shown in Table 1. As can be seen, a clear predominance of women was observed in stones containing apatite and struvite. Cystine stones and other rare types of stones were not included in the further analysis due to their genetic origin and supposed lack of association with environmental factors.

**Table 1:** Distribution of kidney stone types according to gender between 2017- 2018.

Stone composition	Total (N= 1158)	Gender M /F	Age
COM	450 (38.8%)	293 (63.6%) / 157 (36.4%)	49±13
COD	256 (22.1%)	157 (61.8%) / 99 (38.2%)	43±14
COD+HAP	109 (9.4%)	36 (30.3%) / 73 (69.7%)	44±14
HAP	22 (1.9%)	2 (14.3%) / 20 (85.7%)	46±15
Struvite	97 (8.3%)	25 (24.4%) / 72 (75.6%)	44±14
Brushite	21 (1.8%)	17 (89.5%) / 4 (10.5%)	41±12
Uric Acid	129 (11.1%)	103 (78.5%) / 26 (21.5%)	54±12
Uric Acid+ COM	65 (5.6%)	52 (79.7%) / 13 (20.3%)	54±13
Cystine and other rare types	9 (0.8%)	5 (56%) / 4 (44%)	43±22

Age data are reported as mean(SD). COM: calcium oxalate monohydrate; COD: calcium oxalate dihydrate; HAP: apatite.

**Figure 1:**
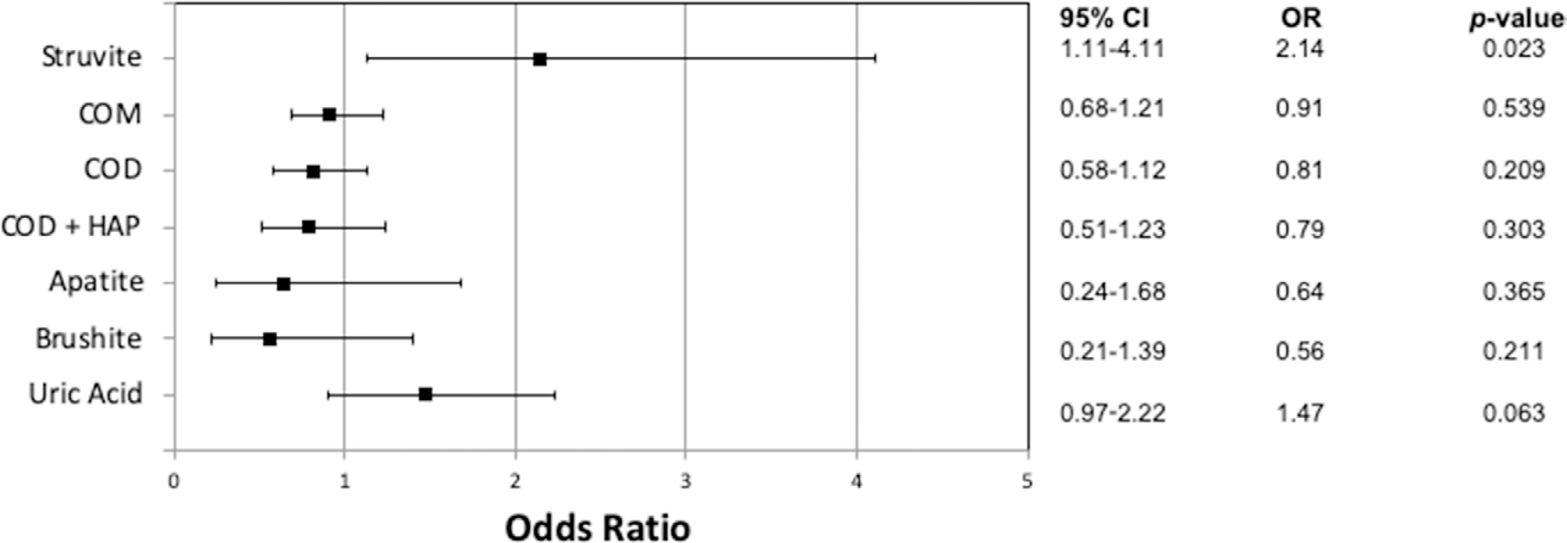
Logistic regression model assessing the impact of low human development index (HDI<0.800) on kidney stone composition in Brazil. COM: calcium oxalate monohydrate; COD: calcium oxalate dihydrate; HAP: apatite.

**Figure 2:**
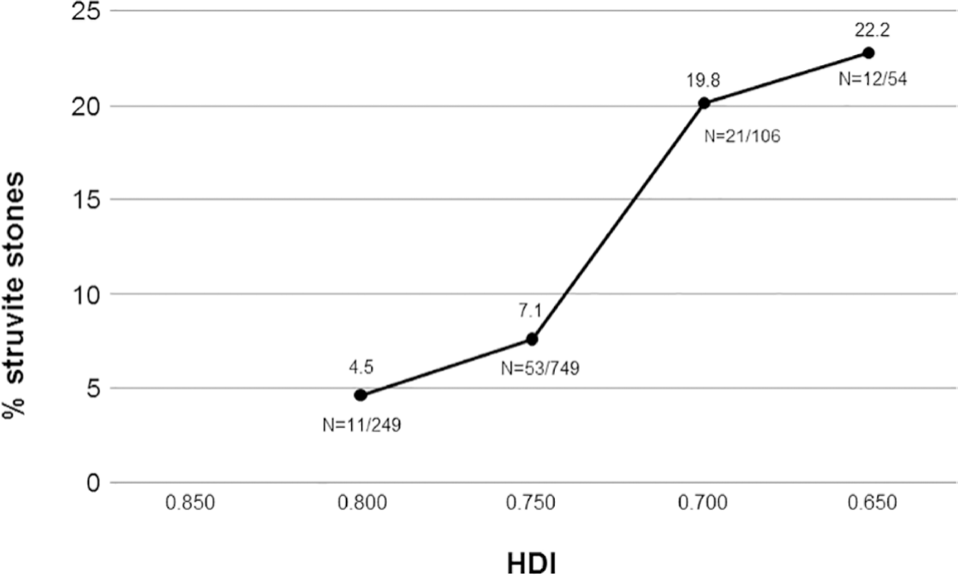
Struvite stones percentages according to human development index (HDI) among stone formers.

In order to assess the potential association between HDI (<0.800) and stone types, gender-adjusted logistic regression models were implemented (Figure 1). People living in cities with a HDI below 0.800 had twice the odds of developing a struvite stone than those living in cities with a higher HDI (OR=2.14 95%, CI 1.11 - 4.11). On the other hand, the HDI was not statistically significant as an independent predictive factor for all other stone compositions. In a further analysis including all other types of stones, we subdivided HDI into four different groups (>0.800; 0.800-0.750; 0.750-0.700; <0.700) and observed a gradual and progressive increase in the percentages of struvite stones, as presented in Figure 2. Reports of more than two episodes of UTI per year were detected in 81.7% of struvite stones compared to only 14.4% among all the remaining stone types (data not shown in tables). Regarding climatic conditions, we created a separated logistic regression for each type of stone using temperature and humidity as independent contributors to the climate and also included gender and HDI level as covariates, which can be seen in Table 2. We did not find any association between the evaluated stone types and mean annual temperature or humidity.

**Table 2:** Separate logistic regression model: stone composition according to temperature, humidity, HDI, and gender distribution.

Stone type	Covariates	Odds Ratio (OR)	95% CI	p value
COM				
	>25°C			0.365
	20-25°C	1.21	0.90-1.64	0.201
	<20°C	1.36	0.79-2.33	0.264
	High Humidity	1.37	0.93-2.02	0.107
	HDI > 0.800	1.24	0.93-2.04	0.409
	Male Gender	1.43	1.12-1.84	0.004
COD				
	>25°C			0.845
	20-25°C	1.02	0.71-1.45	0.930
	<20°C	0.86	0.46-1.59	0.638
	High Humidity	1.29	0.81-2.07	0.279
	HDI > 0.800	1.05	0.59-1.85	0.873
	Male Gender	1.09	0.82-1.46	0.538
Apatite				
	>25°C			0.124
	20-25°C	0.73	0.44-1.22	0.232
	<20°C	1.56	0.59-4.15	0.365
	High Humidity	0.79	0.45-1.40	0.424
	HDI > 0.800	1.53	0.64-3.65	0.339
	Male Gender	0.23	0.15-0.35	<0.001
Struvite				
	>25°C			0.442
	20-25°C	0.97	0.56-1.65	0.904
	<20°C	0.59	0.24-1.42	0.239
	High Humidity	0.75	0.40-1.39	0.360
	HDI > 0.800	0.32	0.12-0.79	0.015
	Male Gender	0.21	0.13-0.34	<0.001
Brushite				
	>25°C			0.701
	20-25°C	0.87	0.26-2.89	0.821
	<20°C	0.46	0.07-2.94	0.417
	High Humidity	0.64	0.17-2.37	0.502
	HDI > 0.800	1.08	0.22-5.31	0.922
	Male Gender	2.92	0.97-8.79	0.056
Uric Acid				
	>25°C			0.715
	20-25°C	0.91	0.62-1.33	0.616
	<20°C	0.75	0.37-1.51	0.424
	High Humidity	0.68	0.44-1.01	0.111
	HDI > 0.800	0.54	0.30-1.14	0.119
	Male Gender	3.27	2.24-4.76	<0.001

Temperature (°C) refers to mean annual temperature. HDI: human development index; COM: calcium oxalate monohydrate; COD: calcium oxalate dihydrate.

## Discussion

Beyond the impact of genetics, diet, age, gender, and BMI, large variations in demographic data between countries might influence the worldwide distribution of urolithiasis and kidney stone composition^13^. Among them, there is climate, seasonal temperature, sunlight exposure, global warming, occupation, urban living (in contrast with rural), socioeconomic background, and access and cost of surgical and clinical therapy, which translate the geographic diversity across the globe^14^^-^ 15.

Moreover, the HDI is a statistic composite index of life expectancy, education, and per capita income indicators, which are used to rank countries and cities and could represent a broader view of some of the aforementioned factors and social disparities. Thus, we hypothesized that it could be related to stone composition as well. Even though a handful of studies have addressed the roles of climate and temperature in the prevalence of stone disease, to the best of our knowledge, this is the first study associating HDI to stone composition. In the present study, calcium oxalate and uric acid stones were the most frequent types, but a high prevalence of struvite stones was perceived in regions of low HDI within the country. Reinforcing our data, developed countries such as Spain^12^, with HDI consistently over 0.800, exhibit much lower rates of struvite stones, around 4.1% when compared to our findings. On the other hand, temperature and humidity did not exhibit an important impact on stone composition in the current sample. In the present series, a higher prevalence of uric acid composition with aging was disclosed, in accordance with other studies^16^. Regarding gender, we observed a male predominance for calcium oxalate and uric acid stones as previously described by Daudon M et al.^13^.

Despite a clear direct association between season and climate, with a higher prevalence of kidney stones, renal colic episodes or number of hospital admissions for urolithiasis treatment in the warmer months of the year^17^, little data focusing on stone composition is available^18^. High daily temperature is considered a risk factor for urolithiasis since it causes water loss and dehydration, resulting in a low urinary volume and pH, which increases urinary saturation for various types of stones^19^^-^ 22. Although some reports did find increases in the risk of stone formation between 10 and 30°C 23, we did not find significant differences concerning the type of formed stone among our different intervals of mean annual temperatures (<20, 20-25, >25°C). Furthermore, for any given climatic condition where humidity is low and the air is dry, more water is lost due to the increased sweating, thus possibly decreasing urine volume and increasing urinary saturation, but the independent effect of humidity is not well established^24^. In the present study, using humidity and temperature separately in our regression model, we did not find statistical difference among all types of stones concerning such parameters. Although not exactly comparable, our findings corroborated Buttigieg et al.^11^, who observed no association between chemical composition and season, and also with a recent study on stone composition within the USA showing very few differences across all states according to climate^18^.

Socioeconomic and other disparities in healthcare have been widely documented in urological practice, in terms of medical prescription, imaging exams, and interventional treatments - even in developed countries^24^^-^ 26. In our study, a gender-adjusted logistic regression model showed a noteworthy and significant impact of low HDI on struvite stones but not for other stone compositions. However, a tendency for more uric acid stones among patients living in cities with lower HDI was observed, in accordance with previous data reporting increased prevalence of uric acid stone in developing areas^27^. Furthermore, the lower the HDI, the higher was the prevalence of struvite stones.

In the current series, we have categorized struvite stones as containing any amount of magnesium ammonium phosphate (MAP) greater than 30%, associated or not with carbapatite, as advocated by others^28^. The percentage of these calculi was nearly 3-fold higher among females than in males among all age groups, in accordance with the literature^29^. Lower social economic status is closely related to lower rates of preventive management and poorer control of acute, chronic, or recurrent conditions such as urolithiasis, which is translated into the associations presently disclosed with HDI. The underlying causes of poor quality of care, particularly for these types of calculi, mostly observed in low HDI areas, indicate delayed or no treatment of recurrent urinary tract infections predisposing the growth of infection stones or even progression to staghorn calculi, and also delay and lesser access to procedures for stone removal and treatment of severe complications^30^.

Limitations of our study include its retrospective design, which does not provide information on dietary habits, lifestyle factors, and biochemical data concerning metabolic disturbances. Episodes of urinary tract infections relied on self-reports and might have been under- or overestimated. Exposure concerning professions, eventual access to air-conditioning, or the particular effect of high temperature in specific populations such as elderly patients could not be accurately assessed.

## Conclusions

Our study demonstrated that patients living in areas with low HDI are more prone to developing struvite stones. Temperature and humidity did not represent a specific risk factor for any stone type in our population.

**Abbreviations**
BMI: Body Mass Index
COM: Calcium Oxalate Monohydrate
COD: Calcium Oxalate Dihydrate
DASH: Dietary Approaches to Stop Hypertension
HAP: Apatite
HDI: Human Development Index
MAP: Magnesium Ammonium Phosphate
UTI: Urinary Tract Infection
